# Integration of Quantitative Trait Loci Mapping and Expression Profiling Analysis to Identify Genes Potentially Involved in Ramie Fiber Lignin Biosynthesis

**DOI:** 10.3390/genes10110842

**Published:** 2019-10-24

**Authors:** Jianrong Chen, Jing Rao, Yanzhou Wang, Zheng Zeng, Fang Liu, Yinghong Tang, Xiaorong Chen, Chan Liu, Touming Liu

**Affiliations:** 1 College of Biological and Environmental Engineering, Changsha University, Changsha 410003, China; z20080849@ccsu.edu.cn (J.C.); lf800825@163.com (F.L.); 2 Institute of Bast Fiber Crops, Chinese Academy of Agricultural Sciences, Changsha 410205, China; leonala@163.com (J.R.); wyzhcf@163.com (Y.W.); 13574363462@163.com (Z.Z.); liuchan@caas.cn (C.L.); 3 College of biological and environmental sciences, Hunan University of Arts and Science, Changde 410128, China; tyhisw1314@163.com; 4 Laboratory of ramie, Yichun Institute of Agricultural Sciences, Yichun 336000, China; C2368457299@163.com

**Keywords:** ramie, lignin content, QTL, gene expression, sequence variation

## Abstract

Ramie fibers, one of the most important natural fibers in China, are mainly composed of lignin, cellulose, and hemicellulose. As the high lignin content in the fibers results in a prickly texture, the lignin content is deemed to be an important trait of the fiber quality. In this study, the genetic basis of the fiber lignin content was evaluated, resulting in the identification of five quantitative trait loci (QTLs). Three genes, *whole_GLEAN_10021050*, *whole_GLEAN_10026962*, and *whole_GLEAN_10009464* that were identified on the QTL regions of *qLC7*, *qLC10,* and *qLC13*, respectively, were found to be homologs of the *Arabidopsis* lignin biosynthetic genes. Moreover, all three genes displayed differential expression in the barks located in the top and middle parts of the stem, where lignin was not being synthesized and where it was being biosynthesized, respectively. Sequence comparison found that these three genes had wide variations in their coding sequences (CDSs) and putative promoter regions between the two parents, especially the MYB gene *whole_GLEAN_10021050*, whose protein had insertions/deletions of five amino acids and substitutions of two amino acids in the conserved domain. This evidence indicates that these three genes are potentially involved in lignin biosynthesis in ramie fibers. The QTLs identified from this study provide a basis for the improvement of lignin content and fiber quality in ramie breeding. The characterization of the three candidate genes here will be helpful for the future clarification of their functions in ramie.

## 1. Introduction

Plant fibers are widespread among vascular plant species and are present in various organs, including leaves, stems, and roots. Fibers are important for plant growth and development because of their functions in establishing plant architecture, defense against herbivores, and storage of ergastic carbon resources and water [1]. Additionally, plant fibers are one of the most important renewable resources and are used as raw material in the paper industry and for the manufacture of various textiles and composites.

Plant fibers include specialized secondary cellular walls (SCWs) that are mainly composed of lignin, cellulose, and hemicelluloses (i.e., xylan and glucomannan) [2]. Lignin is one of the main components of plant fibers, and its biosynthesis and deposition into the SCWs is pivotal for fiber formation. Several enzyme-producing genes involved in lignin biosynthesis and polymerization have been identified in *Arabidopsis*. Their expression has been found to be activated at the right moment and location by a complex regulatory network mainly consisting of the NAC and MYB transcription factors [3]. 

Ramie (*Boehmeria nivea* L.) is one of the world’s oldest fiber crops, and has been cultivated for thousands of years in China [4,5]. Ramie fibers extracted from stem barks possess excellent characteristics, including long strands. The length of a ramie fiber cell can reach up to 55 cm, which is rare among plant fibers [6]. Because of their considerable economic importance, genetic and breeding studies have paid significant attention to the traits of ramie fibers. Using sequence repeat markers, Liu et al. recently constructed the first genetic linkage map of ramie and detected 33 novel fiber-yield-related quantitative trait loci (QTLs) in this crop [7]. More recently, this group also developed a high-density genetic map of ramie containing 4338 single nucleotide polymorphisms (SNPs) and resulting in the identification of 15 fiber-yield-related QTLs. The candidate gene (*qBT4a*) was identified in one of these QTLs [8]. These studies not only remarkably improve our understanding of the quantitative traits of ramie, especially fiber-yield-related agronomic traits, but also provide an essential basis for the improvement of fiber yield traits by marker-assisted selection in this crop.

Lignin is one of the main components of ramie fiber, and its high content in this fiber causes a prickle in the corresponding textile. Thus, the content of lignin is deemed to be an important trait of ramie fiber quality. Many homologs of *Arabidopsis* lignin biosynthetic genes have been identified in ramie [9,10,11,12,13], and their expression has shown a significant association with the accumulation of lignin in ramie [14,15]. However, none of these genes have been proven to be involved in lignin biosynthesis in ramie fibers. The genetic and molecular bases of lignin biosynthesis in ramie fibers are still poorly understood. Recently, a high-density genetic map with 1085 markers spanning 2118.8 cM was constructed using an F_2_ agamous line population derived from two parents, cultivated ramie Zhongsizhu 1 (ZSZ1) and its wild progenitor *B. nivea* var. *tenacissima* (BNT) [16]. Additionally, our previous study characterized the phloem-development-related expression profiling of the stem barks in ramie to identify the differentially expressed genes (unpublished data; sequence reads have been deposited in the SRA database). In this study, we utilized this population to evaluate the genetic basis of the trait of lignin content in ramie fibers; then, the genes located in the QTL region were detected, and their expression level (collected from our previous expression profiling), sequence variation, and function of *Arabidopsis* homolog were analyzed, which will provide an important basis for the identification of candidate genes. 

## 2. Materials and Methods

### 2.1. Experimental Population and Phenotypic Measurements

A ramie F_2_ agamous line population consisting of 111 lines was derived from two parents. We cultivated the ramie Zhongsizhu 1 (ZSZ1) and its wild progenitor *B. nivea* var. *tenacissima* (BNT) according to the method described by Wang et al [16]. All the F_2_ progeny and the two parents were reproduced by cutting propagation and generating an agamous line, which had an identical genotype to the corresponding F_2_ individual. All these agamous family lines and parents were grown on the experimental farm at the Institute of Bast Fiber Crops (IBFC), Changsha, China in June 2016. Two replicates were grown in a randomized complete block design. For each family line, ten seedlings were grown in a plot with two rows, with a distance of 45 cm between the rows and 70 cm between two plants in each row. The area around the population was planted with two-row ramie, which was used to eliminate the boundary effect in the population. The population and two parents were cultivated twice in different periods: the one planted from 10 April 2017 to 14 June 2017 (Environment 1) and another one grown from 15 June 2017 to 4 August 2017 (Environment 2). For each line, the fibers from these stem barks were extracted and dried, and were then used for the estimation of lignin content according to the methods described by Tang et al. [14]. 

### 2.2. QTL Analyses

A high-density genetic map with 1085 markers has been developed and reported previously, spanning a total length of 2118.8 cM [16]. The QTLs of the lignin content trait were detected in two environments using a composite interval mapping with the software MapQTL [17]. The window size was set at a 1 cM threshold significance level and was used to identify the significant markers as cofactors. The experimental likelihood of odd (LOD) threshold significance level was determined by computing 1000 permutations (*p* < 0.05) using a permutation test program (MapQTL) [17].

### 2.3. Identifying the Differentially Expressed Genes (DEGs) Located in the QTL Regions

Elite cultivar Zhongzhu 1 was planted in the experimental farm of the Institute of Bast Fiber Crops, Chinese Academy of Agricultural Sciences, Yuanjiang, China in June 2016. In May 2017, when the ramie grew to 1.5 meters, the barks from the top part of the stem (TPS) and the middle part of the stem (MPS) were separately collected from five individuals according to the description of Chen et al. [18], and immediately frozen in liquid nitrogen. Three replicates were sampled for TPS and MPS barks. Total RNA was extracted using the E.Z.N.A. Plant RNA kit (OMEGA Bio-Tek, Norcross, GA, USA). Total RNAs from six samples were used separately to construct cDNA libraries, and then were sequenced using the Illumina sequencing platform (HiSeq™ 2500; Illumina, San Diego, CA, USA), according to the manufacturer’s instructions. Thereafter, the raw reads for each sample were filtered, generating clean reads used for further analysis (sequence reads deposited in the SRA database under accession no. SRX5776016-SRX5776021). 

To estimate the gene expression level, the clean reads from transcriptome sequencing were aligned to the ramie genome (accession ID: PHNS00000000) using the Bowtie2 software [19], and the read number of each gene was calculated using the RSEM software [20]. The fragments per kilobase of transcript sequence per million base pairs sequenced (FPKM) were estimated to measure the expression of each transcript [21]. The DEGseq program based on the MA-plot algorithm [22] was used to identify the DEGs. For each gene, fold changes and *p*-values adjusted (Q value) for multiple testing with the Benjamini–Hochberg procedure [23] were used to control for false positives. Genes were deemed to have significant differential expression if the Q value obtained was less than 0.05, and there was at least a two-fold change (>1 or <−1 in log2 ratio value that was calculated by the average FPKM value of three MPS libraries divided by that of three TPS libraries.

To obtain the DEGs located in a QTL region, the genes in the QTL region needed to be identified first. The flanking markers of each QTL were assigned to the ramie genome [16,24,25]. In case of a wave in the QTL location by statistical mapping, the genomic region of each QTL was enlarged by outer extending 300 kb from the two flanking markers. The genes in each QTL region (including the QTL interval and the outer extending regions) were identified for the subsequent analysis. The barks from the top and middle parts of the stem, where the SCWs of the fiber cells did not initiate growth, were thickened; thus, the genes involved in fiber formation (including lignin biosynthetic genes) theoretically had differential expression in these two tissues. Finally, the DEGs were searched against the genes identified from the QTL region to obtain the DEGs located in the QTL regions.

### 2.4. DNA Extraction, Sequencing, and Sequence Comparison

DNA was extracted from the fresh leaves of two parents (ZSZ1 and BNT) using a DNA extraction kit (Tiangen, Beijing, China). The coding sequence and its upstream 2 kb sequence for each gene was amplified from these two parents using a standard PCR protocol with gene-specific primers (Supplemental Table S1). For sequencing, 8 μL of the PCR products corresponding to each parent were digested using 5 U of *Exo*I (NEB) and 0.13 U of shrimp alkaline phosphatase (Fermentas) and sequenced using a 3730xl DNA Analyzer (ABI, USA). The sequence contigs were assembled using SEQUENCER 4.1.2 (Gene Codes Co., Ann Arbor, USA), and the assembled three gene cDNA sequences of the two parents were submitted to GenBank along with the accession numbers MN326801–MN326806. The sequence alignment of the DNAs and proteins of these three genes was performed between ZSZ1 and BNT using Clustal Omega [26] and Muscle [27]. The conserved protein-encoding domain was identified using the NCBI conserved domain database [28].

## 3. Results

### 3.1. QTL Mapping for the Lignin Content Trait

The two parents, ZSZ1 and BNT, had a considerable difference in the fiber lignin content. The lignin content in the ZSZ1 fibers was >10%, which was more than 3-fold that in the BNT fibers (<3%, Table 1). A significant variation was observed for this trait between the two growing environments (Table 1).

**Figure 1:**
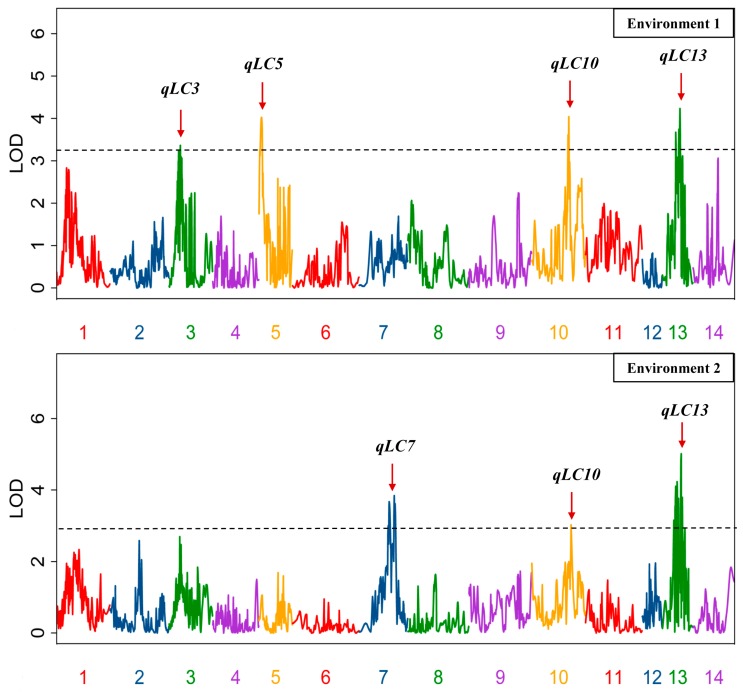
QTLs for the trait of lignin content in the fibers. The broken line indicates the genome-wide significance likelihood of odd (LOD) threshold, and the red arrow indicates the LOD peak of the QTLs.

Using genotype data of the population [16], the QTLs for the trait of fiber lignin content was detected. The LOD thresholds (1000 permutations) were estimated for the investigated traits in the two environments, and the values were 3.25 and 2.98, respectively. Based on these thresholds, a total of four and three QTLs were identified for the fiber lignin content trait in Environment 1 and Environment 2, respectively; they accounted for the population variation ranging from 16.1% to 23.4%. Three and one QTL were observed with over-dominance in Environments 1 and 2, respectively (Table 2, Figure 1). Two QTLs (*qLC10* and *qLC13*) were identified in both environments, resulting in a total of five QTLs. All five QTLs increased the fiber lignin content by the ZSZ1 allele, and three QTLs (*qLC3*, *qLC5*, and *qLC10*) showed over-dominance (Table 2). 

### 3.2. Detecting DEGs Located in the QTL Regions

**Figure 2:**
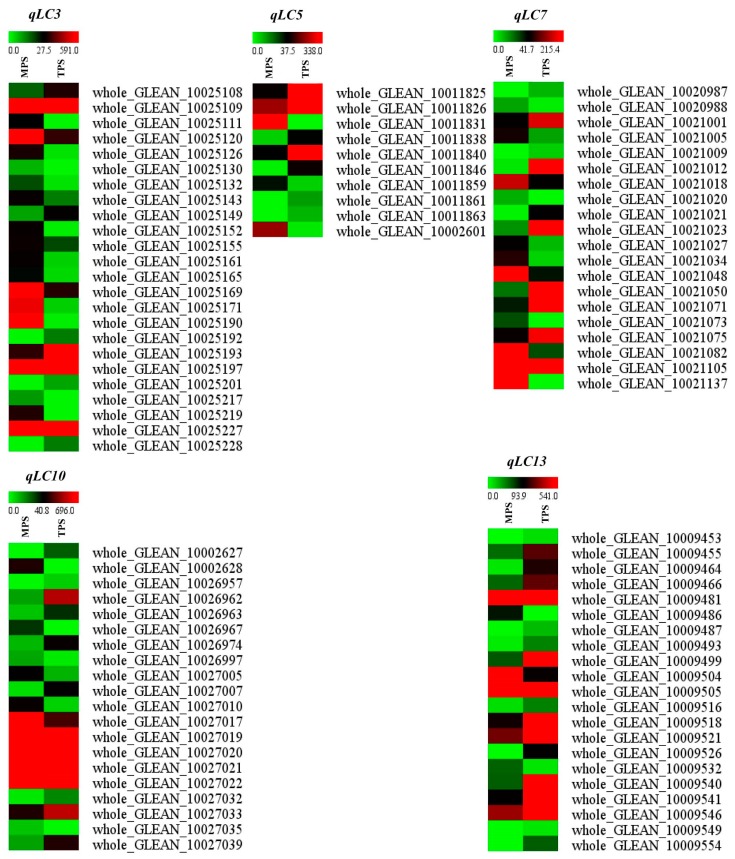
Differentially expressed genes in the QTL region. TPS and MPS indicate the gene expression level in the barks located at the top and middle parts of the stem, respectively.

After anchoring the flanking markers of the QTL into the ramie genome, the genomic regions of the five QTLs were identified. *qLC3* was located in the scaffold PHNS01003751.1, *qLC5* in PHNS01005483.1 and PHNS01000277.1, *qLC7* in PHNS01007792.1, *qLC10* in PHNS01007780.1 and PHNS01006792.1, and *qLC13* in PHNS01000489.1. A total of 156, 122, 190, 152, and 167 genes were located in the QTL regions *qLC3*, *qLC5*, *qLC7*, *qLC10,* and *qLC13*, respectively. Of these, 24, 10, 20, 20, and 21 genes showed differential expression between the barks located at the top and middle parts of the stem, where the SCWs of the fiber cells did not initiate growth and were thickened, respectively (Table S2, Figure 2). This finding suggests that these genes are potentially involved in the biosynthesis of the fiber SCWs, including lignin biosynthesis. 

### 3.3. Identifying the Differentially Expressed Genes that Are Homologous with *Arabidopsis* Lignin Biosynthetic Genes from the QTL Regions 

**Figure 3:**
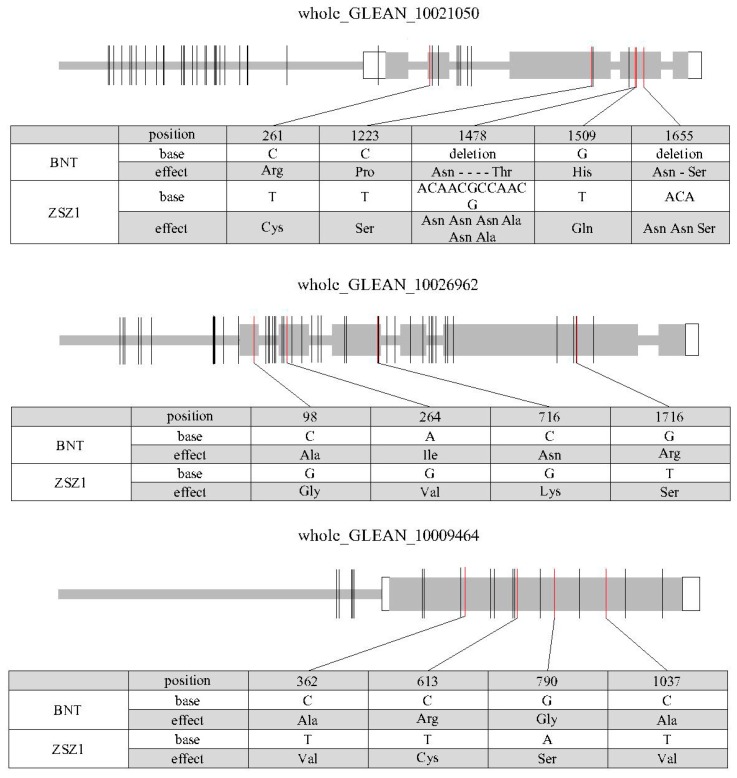
Sequence variation of the three candidate genes in the two parents, BNT and ZSZ1. The gray and white rectangles indicate the coding sequence (CDS) and untranslated region (UTR), respectively. The thick gray lines before the UTR and in the inter-exon region represent the putative promoter and intron region, respectively. The vertical molding in the gene indicates variation in the corresponding position of a gene, and the red one in the CDS represents variation that caused the change in an amino acid. The tables under each figure show the detailed sequence variations in CDS and the corresponding amino acid change for each gene.

*HCT* and *LAC17*, encoding shikimate hydroxycinnamoyl transferase and laccases, respectively, are two important lignin biosynthetic genes in *Arabidopsis* [29,30]. The MYB transcription factor plays key roles in plant growth and development, including in regulating lignin biosynthesis [31]. In this study, three differentially expressed genes—*whole_GLEAN_10021050*, *whole_GLEAN_10026962*, and *whole_GLEAN_10009464*—were identified from the QTL regions *qLC7*, *qLC10,* and *qLC13*, respectively. *whole_GLEAN_10021050* encoded an MYB protein and the other two genes were homologs of the *Arabidopsis LAC17* and *HCT* genes. The transcripts of these three genes were collected from the ramie transcriptome (i.e., T4_Unigene_BMK.18380 comp47560_c0 and comp44336_c0 for *whole_GLEAN_10021050*, *whole_GLEAN_10026962*, and *whole_GLEAN_10009464*) [32], suggesting that these three genes predicted from the genome were authentic. Next, the sequences of these transcripts were aligned with the genome to determine their gene structures, which revealed that there were five, six, and one exon in *whole_GLEAN_10021050*, *whole_GLEAN_10026962*, and *whole_GLEAN_10009464*, respectively (Figure 3).

### 3.4. Sequence Comparison of the Three Identified Lignin Biosynthesis Gene Homologs

The sequence variation of the three DEGs identified was further characterized by sequence comparison with the genes of the two parents, ZSZ1 and BNT. In the MYB gene *whole_GLEAN_10021050* from *qLC7*, there was a 12-bp deletion and a 3-bp deletion in the fourth exon of the BNT allele, and one insertion variation each in the putative promoter and intron region of the BNT allele. A total of 27, 1, 7, and 4 SNPs were identified in the putative promoter, untranslated regions (UTRs), coding sequence (CDS), and intron region, respectively. The variations in the CDS region caused the substitution of seven amino acids and the deletion of five amino acids in the whole_GLEAN_10021050 protein of wild BNT, including two amino acids that fell into the conserved DNA binding domain of the MYB transcription factor (Figure 3). In the *LAC17* homolog *whole_GLEAN_10026962* from *qLC7*, there were 5 SNPs and 3 insertion/deletion variations identified in the putative promoter and 16 variations in the intron region. A total of 21 SNPs were found in the CDS region, which resulted in the substitution of four amino acids in the putative protein (Figure 3). In the *HCT* homolog *whole_GLEAN_10009464*, 3 SNPs and 3 insertion/deletion variations were identified in the putative promoter, and 15 SNPs in the CDS region that caused the substitution of four amino acids in the putative protein (Figure 3).

## 4. Discussion

### 4.1. Genetic Basis for the Trait of Lignin Content in Ramie Fibers

Lignin is one of three main components of ramie fiber, and it is an important factor in determining fiber quality. The trait of lignin content in ramie fibers is inherited quantitatively and is typically controlled by several major and minor QTLs. In recent years, genetic studies on the quantitative traits of ramie have considerably progressed, and several genetic maps have been constructed [7,8,16]. Scores of QTLs for fiber yield traits have been detected [7,8,33], providing an opportunity to characterize the lignin content trait at the genetic level. In this study, five QTLs for the trait of fiber lignin content were first identified. Notably, there were many fiber yield and flowering time-related QTLs with over-dominance, and these QTLs were considered to be related to the heterosis of the corresponding traits [7,33]. In this study, three of the five QTLs were found to have over-dominance, suggesting that these three QTLs may contribute to the heterosis of the lignin content traits. Presently, QTLs with over-dominance have been identified for all the investigated traits, indicating that the over-dominant loci are likely to be universal in the ramie genome. Ramie is heterozygous under natural conditions, and no homozygous ramie plants have been reported until now, because ramie that has self-crossed for several generations cannot survive due to the severe inbreeding depression. Therefore, this study, for the first time, uncovered the genetic basis of the lignin content trait. Identification of the associated QTLs provided a basis for the improvement of lignin content and fiber quality in ramie breeding.

### 4.2. Potential Candidate Genes

Plant fibers are mainly composed of lignin, cellulose, and hemicelluloses (i.e., xylan and glucomannan); thus, fiber formation mainly involves the biosynthesis of lignin, cellulose, and hemicellulose. In *Arabidopsis*, approximately 80 genes have been identified to be involved in fiber formation [3], including the lignin biosynthetic genes *HCT* and *LAC17* [29,30]. Because many genes are involved in the process of fiber formation, they need to be expressed at the right moment and location. This coordinated activation of the genes is controlled by a transcriptional network involving NAC and MYB master switches and their downstream transcription factors [3,31]. In the transcriptional network of *Arabidopsis*, at least 10 NAC genes are top-level master switches regulating the expression of second-level master switches. *MYB46*, *MYB83*, and *MYB46*/*MYB83* modulate the expression of downstream transcription factors, precisely controlling the entire SCW biosynthetic program [3]. Presently, at least 16 MYB proteins have been found to be involved in this transcriptional network. These MYB proteins have key roles in fiber formation, including lignin biosynthesis. 

Although transcriptome analysis provided new insights into ramie fiber formation [18,34], and many ramie NAC and MYB genes have been identified from the transcriptome [35,36], the mechanism of fiber formation (including the lignin biosynthetic mechanism) is still unclear. In the present study, three genes, *whole_GLEAN_10021050*, *whole_GLEAN_10026962*, and *whole_GLEAN_10009464*, were identified as being potentially involved in lignin biosynthesis in ramie fibers, based on the following evidence. First, these three genes were located in the lignin content QTL region. Second, they encode the MYB protein, shikimate hydroxycinnamoyl transferase, and laccases, respectively, and are homologs of *Arabidopsis* lignin biosynthetic genes. Third, all three genes show differential expression in two different stages of fiber development. Finally, all three genes show a large variation in their CDS and putative promoter region between two parents, especially the MYB gene *whole_GLEAN_10021050*, whose protein has insertion/deletion of five amino acids and two amino acid substitutions inside the conserved domain of the MYB protein. Identification of these three candidate genes provides an important basis for the future clarification of their functions in ramie lignin biosynthesis. 

## 5. Conclusions

In this study, the genetic basis of the trait of lignin content in ramie fibers was assessed, resulting in the identification of five QTLs. This provides a basis for the improvement of lignin content and fiber quality in ramie breeding. Additionally, several pieces of evidence revealed that three genes, *whole_GLEAN_10021050*, *whole_GLEAN_10026962*, and *whole_GLEAN_10009464,* could possibly be involved in lignin biosynthesis in ramie fibers. Identification of these candidate genes provides an important basis for the future clarification of their functions in lignin biosynthesis in ramie fibers. 

## Figures and Tables

**Table 1:** Descriptive statistics of the trait of fiber lignin content for the parents and the population.

Environment	Population		Parents	
	Range (%)	Mean ± SD (%)	ZSZ1 (%)	BNT (%)
Environment 1	1.95–12.09	5.55 ± 2.07	10.91	2.97
Environment 2	1.96–10.20	4.54 ± 1.63	10.27	2.73

BNT: *Boehmeria nivea* var. *tenacissima* (wild progenitor of ZSZ1); ZSZ1: cultivated ramie Zhongsizhu 1; SD: standard deviation.

**Table 2:** Quantitative trait loci (QTLs) identified for the trait of fiber lignin content from ZSZ1/BNT populations in two environments.

Environment	QTL	Linkage Group	Interval	LOD ^a^	Add ^b^ (%)	Dom ^c^ (%)	D/A ^d^	Var% ^e^
Environment 1	*qLC3*	3	Marker_4740–Marker_967	3.36	1.31	−1.55	−1.18	19.1
	*qLC5*	5	Marker_4067–Marker_213	4.02	1.22	−2.11	−1.73	22.4
	*qLC10*	10	Marker_317–Marker_1605	4.04	1.08	1.32	1.22	22.5
	*qLC13*	13	Marker_5976–Marker_1274	4.23	1.13	−0.18	−0.16	23.4
Environment 2	*qLC7*	7	Marker_5787–Marker_4916	3.84	0.86	−0.72	−0.84	16.1
	*qLC10*	10	Marker_317–Marker_1605	3.02	0.90	1.16	1.29	12.9
	*qLC13*	13	Marker_5976–Marker_1274	5.01	0.94	−0.13	−0.14	20.4

^a^ Likelihood of odd. ^b^ Additive effect; positive additive effect indicates that the ZSZ1 allele increased the trait. ^c^ Dominance effect. ^d^ The ratio of dominance effect to additive effect. ^e^ Percentage of total phenotypic variance explained by the QTL.

## Supplementary Materials

The following are available online at https://www.mdpi.com/2073-4425/10/11/842/s1, Table S1: Primers for amplifying three candidate genes and their putative promoters, Table S2: Differentially expressed genes in the QTL region.
